# The Changes Produced in the Blood by the Administration of Cod Liver Oil and Cocoa-Nut Oil

**Published:** 1855-09

**Authors:** Theophilus Thompson

**Affiliations:** Physician to the Consumption Hospital at Brompton


The Changes Produced in the Blood by the Administration of Cod Liver Oil and Cocoa-Nut Oil. By Dr. Theophilus Thompson, F. R. S., Physician to the Consumption Hospital at Brompton.

The author has found that during the administration of cod-liver oil to phthisical patients their blood grew richer in red corpuscles, and he refers to a previous observation of Dr. Franz Simon to the same effect. The use of almond-oil and of olive-oil was not followed by any remedial effort; but from cocoa-nut-oil, results were obtained almost as decided as from the oil of the liver of the cod, and the anthor believes it may turn out to be a useful substitute. The oil employed was a pure cocoa oleine, obtained by pressure from crude cocoa-nut-oil, as expressed in Ceylon and on the Malabar coast from the Copperah or dried cocoa-nut kernel, and refined by being treated with an alkali, and then repeatedly washed with distilled water. It burns with a faint blue flame, showing a comparatively small proportion of carbon, and is undrying. The analysis of the blood was conducted by Mr. Dugald Campbell. The whole quantity abstracted having been weighed, the coagulum was drained on bibulous paper for four or five hours, weighed and divided into two portions. One portion was weighed, and then dried in a water-oven, to determine the water. The other was macerated in cold water until it became colorless, then moderately dried, and digested with ether and alcohol, to remove fat; and, finally, dried completely and weighed as fibrin. From the respective weights of the fibrin, and the dry clot, that of the corpuscles was calculated. The following were the results observed in seven different individuals affected with phthisis in different stages of advancement:

| | | Red Corpuscles. | Fibrin |
|---|---|---|---|
| First stage, before the use of cod-liver oil, | Female, | 129·26 | 4·52 |
| | Male, | 116·53 | 13·57 |
| First stage, after the use of cod-liver oil, | Female, | 136·47 | 5·00 |
| | Male, | 141·53 | 4·70 |
| Third stage, after the use of cod-liver oil, | Male, | 138·47 | 2·23 |
| Third stage, after the use of cocoa-nut oil, | Male | 139·95 | 2.31 |
| | Male | 144·94 | 4.61 |

[*Virginia Med. and Surg. Jour.*

---

Syrup of Guaiac.—M. Mouchon (*Bull. Gén. de Thérap.*,) gives several formulæ for preparations of guaiac resin. The base of these is a tincture made with one part of guaiac to four parts of alcohol (fifty-six per cent.) by displacement. To make the syrup of guaiac, half a pound of this tincture is mixed with a pound of syrup of gum arabic, and the alcohol distilled or evaporated off till the syrup weighs one pound.

Thus obtained, syrup of guaiac is very homogeneous, owing to the emulsionizing effect of the gum in suspending the resinous particles.

